# Scleroderma-associated thrombotic microangiopathy in overlap syndrome of systemic sclerosis and systemic lupus erythematosus: A case report and literature review

**DOI:** 10.1097/MD.0000000000022582

**Published:** 2020-10-09

**Authors:** Xiaodong Xie, Guoqin Wang, Hong Cheng, Lijun Sun, Hongrui Dong

**Affiliations:** Department of Renal Division, Beijing Anzhan Hospital, Capital Medical University, Beijing, China.

**Keywords:** overlap syndrome, scleroderma renal crisis, systemic sclerosis, thrombotic microangiopathy

## Abstract

**Rationale:**

Systemic sclerosis (SSc) is a serious multisystem connective tissue disease. When SSc is accompanied by systemic lupus erythematosus (SLE), called SSc-SLE overlap syndrome. SSc associated thrombotic microangiopathy (SSc-TMA) can lead to scleroderma renal crisis, it mainly manifests hypertension or even malignant hypertension, acute kidney injury, and higher mortality. The case of SSc-SLE overlap syndrome combined with SSc-TMA has rarely been reported.

**Patient concerns:**

We report the case of an elderly male with SSc-SLE overlap syndrome combined with scleroderma renal crisis and SSc-TMA.

**Diagnoses:**

The patient has typical of SSc on the face and hands, combined with pulmonary artery hypertension, interstitial lung disease, heart failure and malignant hypertension, as well as SLE, lupus nephritis class V, and TMA, which were definitively diagnosed by clinical laboratory examination and renal histopathology.

**Interventions:**

The patient was treated with prednisone, cyclophosphamid, renin-angiotensin system inhibitors, diuretics, and acetylcysteine.

**Outcomes:**

The patient died suddenly of heart failure on the 35th day after discharge.

**Lessons:**

The occurrence of TMA leads to the deterioration of the prognosis of SSC-SLE overlap syndrome. The diagnosis of SSC-TMA in SSc-SLE overlap syndrome depends on clinical laboratory examination and renal histopathology.

## 1 Introduction

Systemic sclerosis (SSc), also called scleroderma, is a chronic, and complex connective tissue disease (CTD) that is characterized by microvascular endothelial cell damage and progressive fibrosis of the skin and visceral organs (lungs, heart, and kidney).^[1,2]^ When severe acute renal injury occurs, also called scleroderma renal crisis (SRC), it mainly manifests as proteinuria, hematuria, hypertension or even malignant hypertension, and acute kidney injury (AKI). According to the histopathological characteristics, SSc can be divided into 2 types: narrowly defined SRC (nd-SRC) and SSc-associated thrombotic microangiopathy (SSc-TMA). Once SRC occurs, especially SSc-TMA, the mortality associated with SSc is high.^[3]^ SSc predominantly occurs in young and middle-aged women. The ratio of the incidence in men and women is approximately 1:4.7. Approximately 6.8% to 14.7% of SSc patients have overlapping systemic lupus erythematosus (SLE), called SSc-SLE overlap syndrome.^[1,4,5]^ We report a rare case of severe SSc-SLE overlap syndrome combined with SSc-TMA and multiple organ injury in an elderly male.

## 2 Case report

A 61-year-old male was admitted in November 2019. Seven years prior, the patient's hands began turning white, and he experienced cyanosis with pain in his hands. He reported that his fatigue gradually increased starting 5 years prior. One year prior, the skin on the fingertips of both hands began to break. The patient was treated at a local hospital. His blood pressure was approximately 160/100 mm Hg, urinalysis revealed protein (+1) and blood (+2), his serum creatinine level was 76 μmol/L, his albumin level was 32.8 g/L, and his globulin level was 43.6 g/L. He was positive for ANA (1:640), anti-SM antibody, anti-SSA and anti-Ro-52 antibodies. His C3 level was 0.75 g/L (0.9–1.8), his C4 level was 0.11 g/L (0.1–0.4), and his ESR was 66 mm (0–15). Chest CT showed bilateral pulmonary fibrosis. Three months after treatment at the local hospital, edema began to develop in his lower limbs; this edema worsened gradually and was accompanied by chest tightness, asthmatic suffocation, and an inability to lie flat at night due to dyspnea. At that time, the patient's blood pressure had increased to 220/130 mmHg, his urine protein level was 6.7 g/d, his serum creatinine level had increased from 92 to 299 μmol/L, his albumin level was 28.1 g/L, his globulin level was 35.4 g/L, his LDH level had increased to 486 U/L (114–240), and his NT-proBNP level was markedly elevated at over 35000 ng/L (0–900 ng/L). Echocardiography showed left atrial enlargement and pulmonary artery hypertension (PAH). Methylprednisolone was given at a dosage of 40 to 80 mg/d, and antihypertensive and diuretic therapy regimens were administered. There were no typical symptoms of SLE, such as rash, alopecia, photosensitivity, oral ulcer, or arthralgia.

**Figure 1:**
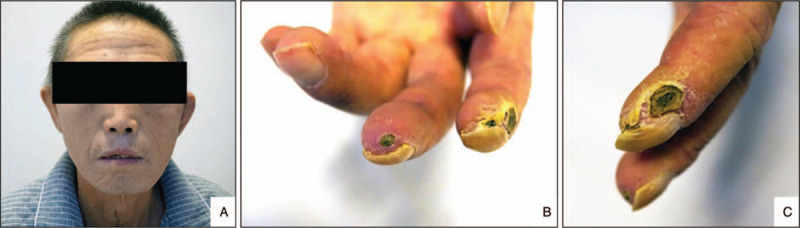
Face and fingers features. A: The facial skin was swollen and stiff with a sharp nose, fewer wrinkles, and atrophic lips. B and C: The fingertips of the forefingers (B, C) and middle fingers (B) showed ulceration with scabs.

The patient's blood pressure was 190/115 mm Hg. His facial skin was swollen and stiff, with a sharp nose, fewer wrinkles and atrophic lips (Fig. 1A). Both hands were swollen and stiff, and the fingertips of the forefingers and middle fingers showed ulceration with scabs (Figs. 1B and 1C). No damage was found on the trunk or upper limbs. Slight symmetrical depressed edema could be seen in both lower limbs.

**Figure 2:**
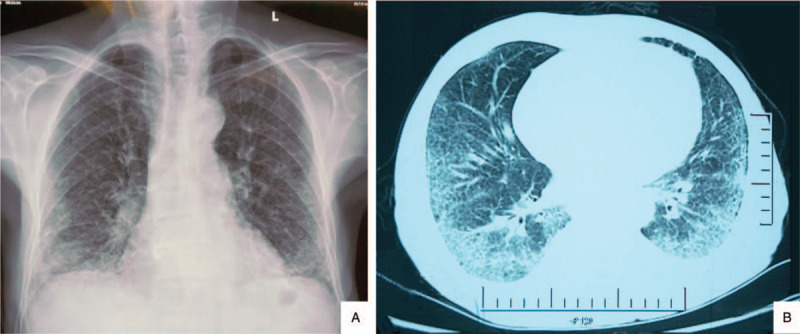
Imaging features. Chest X-ray(A) and CT(B) showed pulmonary interstitial fibrosis.

Routine blood tests showed that his white blood cell count was 12.83 × 10^9^/L, his hemoglobin level was 8.7 g/dL, his platelet count was 135 × 10^9^/L, and his reticulocyte percentage was 2.28% (0.5–1.5). Broken red blood cells were present in peripheral blood smears. His urine protein level was (+3), the urine sediment showed deformed hematuria, and his 24-hour urine protein level was 9.7 g. His serum creatinine level was 337.1 μmol/L, his albumin level was 27.2 g/L, his globulin level was 21.8 g/L, his TG level was 2.2 mmol/L (0–1.7), his TCHO level was 6.99 mmol/L (3.1–5.2), and his UA level was 523 μmol/L (208.3–428.4), with normal serum ions and hsCRP. Arterial blood gas analysis showed hypoxemia. The serum levels of IgA, IgG and IgM were normal; his ESR was 20 mm; his C3 level was 0.64 g/L (0.7–1.4); and his C4 level was normal. His ANA titer was 1:1000 and he was weakly positive for anti-SSA antibodies and strongly positive for anti-Ro-52 antibodies; however, he was negative for anti-dsDNA, anti-SM, anti-SSB, anti-RNP, anti-ScL-70, anti-GBM, and ANCA. His level of serum RF was normal, and the tests for aCL, anti-β2-gp1 and lupus anticoagulant were all negative. Coombs’ test was weakly positive. Chest X-ray and CT showed pulmonary interstitial lesions (Figs. 2 A and 2B). Echocardiography revealed severe pulmonary hypertension and double atrial enlargement (right atrium was 34 × 51 mm) with an EF of 61%. Pulmonary function tests showed a normal ventilation function but a severe decline in diffusion function. Serum protein electrophoresis and serum and urine immunofixation electrophoresis were normal, and there was no visible monoclonal immunoglobulin. Grade III hypertensive retinopathy could be seen on fundoscopy.

**Figure 3:**
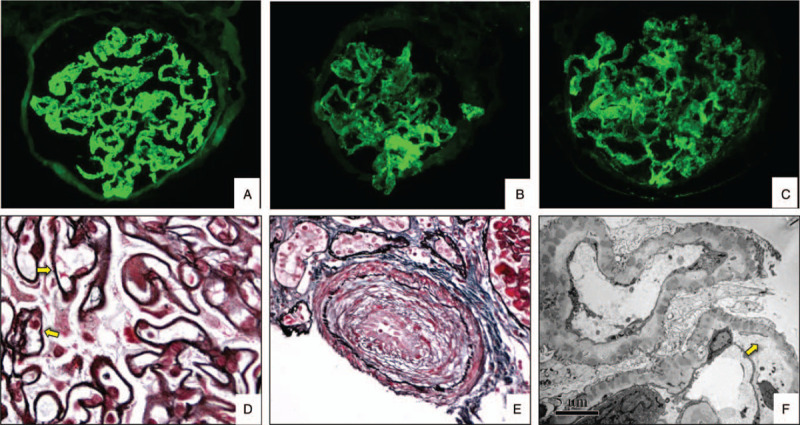
Pathological findings of the renal biopsy. A-C: immunofluorescent staining show IgG(A), C1q(B) and C3(C) deposition in mesangial and capillary wall. D: deposition of immune complexes in the subepithelial space(arrows) (PASM+MASSON). E: interlobular artery lumen was narrow to occlusion due to mucinous edema and endometrial fibrosis (“onion bulbs”) (PASM+MASSON). F: electron microscopy: the loose layer of the glomerular basement membrane was widened (arrow). Original magnification, (A)-(C) × 200, (D) and (E) × 400, (F) × 6000.

Renal biopsy was also performed (Fig. 3). The results confirmed the diagnosis of lupus nephritis (LN) class V (Fig. 3A, B, C, D) and TMA. It was observed that the walls of the renal arterioles were thickened, especially those of the interlobular arteries, which generally had mucinous edema and endometrial fibrosis resembling “onion bulbs” (Fig. 3E), and the lumen was narrowed to the point of occlusion. The loose layer of the glomerular basement membrane was widened, as observed on electron microscopy (Fig. 3F).

The final diagnoses were SSc-SLE overlap syndrome, SRC (SSc-TMA), lupus nephritis class V, malignant hypertension, PAH, congestive heart failure, and hemolytic anemia. The patient was treated with prednisone (40 mg/d), cyclophosphamide (50 mg/d), renin-angiotensin system (RAS) inhibitors, diuretics, and acetylcysteine. After a month of therapy, the patient's blood pressure had decreased to below 140/90 mm Hg, his serum creatinine level had decreased to 293.0 μmol/L and his albumin level had increased to 31.1 g/L. His urinary protein and NT-proBNP levels did not change. Ultimately, the patient died suddenly of heart failure on the 35^th^ day after discharge.

Informed written consent was obtained from the patient for the publication of this case report and the accompanying images.

## 3 Discussion

SSc is a serious multisystem CTD characterized by vasculopathy and fibrosis. Compared with other CTDs, it is associated with a higher mortality rate and can lead to a serious decline in quality of life. SSc can progress through 3 phases: the edematous, indurative and atrophic phases. Initial presentations in the edematous phase included puffy hands, sclerodactyly, and/or Raynaud's phenomenon. Skin tightness is the classical presentation in the indurative phase. According to the severity of skin damage, SSc can also be classified as limited cutaneous SSc (lcSSc) and diffusely cutaneous SSc (dcSSc). lcSSc predominantly affects the peripheral areas of the body distal to the elbows and knees.^[1,2]^ Conversely, dcSSc involves the more proximal aspects of the extremities and the trunk. This patient presented with thickening of the skin on his hands and face, followed by digital ischemia and digital ulcers and scabs, which were also accompanied by Raynaud's phenomenon. His facial skin was stiff, with lip atrophy. There were no abnormalities in the other limbs or trunk. His skin damage, which had progressed to the phase of induration and atrophy, was consistent with lcSSc. Once SSc is accompanied by other CTD features, scleroderma overlap syndrome can be diagnosed. Among the scleroderma overlap syndromes, SSc-polymyositis overlap syndrome is the most common, followed by SSc-SLE overlap syndrome and SSc-rheumatoid arthritis overlap syndrome.^[6]^ At present, there are few large-sample studies of SSc-SLE overlap syndrome. A multicenter study conducted in Canada included 1252 SSc patients, 86 of whom had SSc-SLE overlap syndrome. The prevalence was only 6.8%, and the patients were mostly female. Compared with SSc, SSc-SLE overlap syndrome occurred in younger patients, had a lower incidence of dcSSc, had a higher positivity rate for antiphospholipid antibody, was associated with a longer median survival time, and involved a higher incidence of PAH. There were no significant differences in the incidences of SRC, interstitial lung disease, and digital ulcers.^[4]^ According to the Classification Criteria for SSc 2013 ACR/EULAR (total score >9), this patient was definitively diagnosed with a total score of 12 (sclerodactyly of the fingers: 4, fingertip pitting scars: 3, pulmonary arterial hypertension and/or interstitial lung disease: 2 and Raynaud phenomenon: 3).^[7]^ Meanwhile, based on positivity for ANA and anti-Sm antibodies, a mild decrease in C3, and kidney injury, SLE was also diagnosed, along with histologic lesions typical of LN V observed on renal biopsy.^[8]^ Therefore, the patient was diagnosed with SSc-SLE overlap syndrome.

The incidence of PAH in SSC is higher than that in SLE, at 7% to 12% and 1% to 5%, respectively, accounting for 60% to 80% and 15% to 20% of CTD-associated PAH. SSc-PAH patients have a high mortality rate because they receive no additional benefits from immunosuppressive therapy. The occurrence of PAH with SLE is always associated with the activity of disease, which may be reduced by immunosuppressive therapy.^[9]^ Therefore, we believe that this patient's PAH was more likely to have been related to SSc and not SLE, because his SLE did not involve active lesions. His severe PAH was of a prolonged duration, which led to right heart failure.

SRC is a life-threatening complication commonly associated with dcSSc, and it usually occurs in the first 3 years.^[10,11]^ Our patient developed SRC after 7 years. The initial symptoms of nd-SRC are prominently severely elevated blood pressure, hypertensive retinopathy, and decreased renal function, followed by mild thrombocytopenia. Conversely, SSc-TMA, a severe type of SRC, first presents as severe thrombocytopenia and then as elevated blood pressure and renal function deterioration.^[3]^ Due to the patient's long disease course, the clinical manifestations were increased blood pressure, deteriorated renal function, mild anemia with an increased reticulocyte percentage, visible schizocytes in the peripheral blood smear, and transiently elevated LDH level. Although transient thrombocytopenia was not observed, we made a clinical diagnosis of microangiopathic hemolytic anemia (MAHA). Because of the weak positivity on the Coombs test, we could not exclude hemolytic anemia caused by SLE. Based the clinical data, the likely diagnosis was nd-SRC. The pathology of the kidney biopsy presented as TMA both in the renal arterioles and the glomeruli. It is generally recognized that this pathological disease, similar to TMA associated with SLE, is more common in the active type of LN IV.^[12]^ Therefore, based on the combination of the renal pathology and clinical characteristics, the diagnosis of SSc-TMA was clear, and AKI was also diagnosed. The case we present here was concurrent nd-SRC and SSc-TMA. We inferred that these 2 types may also be different stages of SRC. From the perspective of pathogenesis, SSc can directly lead to TMA.^[13]^ It has also been reported that the treatment of SSc using large doses of steroids or cyclosporine can lead to SSc-TMA.^[3,14]^

To our knowledge, there are only 4 cases of SSc-SLE overlap syndrome combined with SSc-TMA published in English. The characteristics of these cases, including those in the current report, are summarized in Table 1. Unlike in previous reports,^[14–17]^ in which the patients were young women, our patient was an elderly man, and the diagnosis of TMA was confirmed by not only light microscopy but also electron microscopy. The clinical manifestations in the patients were similar, and all had AKI except 1 young woman. In previous case reports, therapy with corticosteroids and immunosuppressants, such as methotrexate, azathioprine or cyclophosphamide, and other regimes for SLE and SSc-SLE overlap syndrome, were preferred,^[14–17]^ and we prescribed a similar treatment regime for this patient. It has been reported that some severe SSc-TMA patients experienced relief with plasma exchange therapy, suggesting that the complement system may be active in the pathogenesis of TMA through the classical pathway. Hence, a regimen involving eculizumab, a C5 suppressor agent, could also be used. The activation of the RAS and endothelin system may also participate in the pathogenesis of SSc. RAS inhibitors and endothelin receptor antagonists are not only effective for the treatment of hypertension but also for the prolongation of survival in SSc patients.^[2,6]^

**Table 1:**
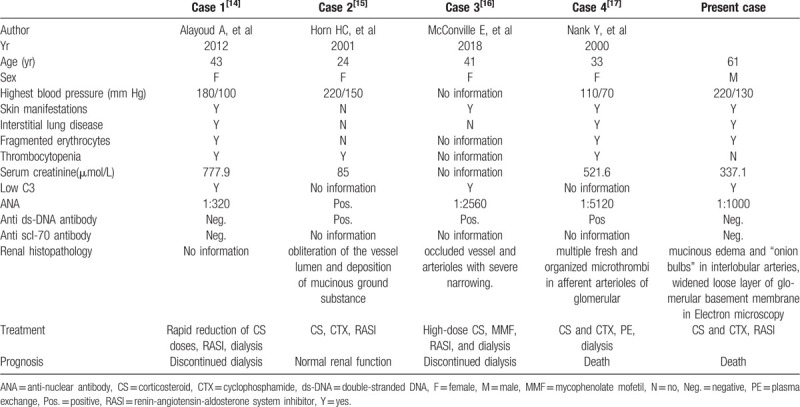
Reported cases of thrombotic microangiopathy in overlap syndrome of systemic sclerosis and systemic lupus erythematosus.

Based on the reports in the literature, the overall prognosis of SSc-TMA is poor. The patient we report here had a long disease course, and at admission, many tissues and organs had already been damaged, such as the skin, lungs, heart and kidneys, which is characteristic of SSc-TMA overlap syndrome. Although we prescribed corticosteroids and immunosuppressant treatment, and the patient's renal function slightly improved, he still died soon after discharge. It is necessary to improve the early recognition and diagnosis of overlap syndrome to improve the survival of patients with this disease.

## Author contributions

**Conceptualization:** Hong Cheng.

**Data curation**: Xiaodong Xie, Guoqin Wang.

**Resources**: Lijun Sun, Hongrui Dong.

**Writing – original draft**: Xiaodong Xie, Guoqin Wang.

**Writing – review & editing:** Hong Cheng

## Abbreviations

Abbreviations: AKI = acute kidney injury, dcSSc = diffusely cutaneous SSc, nd-SRC = narrowly defined SRC, lcSSc = limited cutaneous SSc, RAS = renin-angiotensin system, SLE = systemic lupus erythematosus, SSc = systemic sclerosis, TMA = thrombotic microangiopathy, SRC = scleroderma renal crisis.
